# Supplementary material for: Fused Cells between Human-Adipose-Derived Mesenchymal Stem Cells and Monocytes Keep Stemness Properties and Acquire High Mobility
Source: Int J Mol Sci. 2022 Aug 26;23(17):9672. doi: 10.3390/ijms23179672 (PMC9456160; doi:10.3390/ijms23179672)
Supplement: Supplementary file 1 [file ijms-23-09672-s001.zip › ijms-1835411-supplementary.pdf]

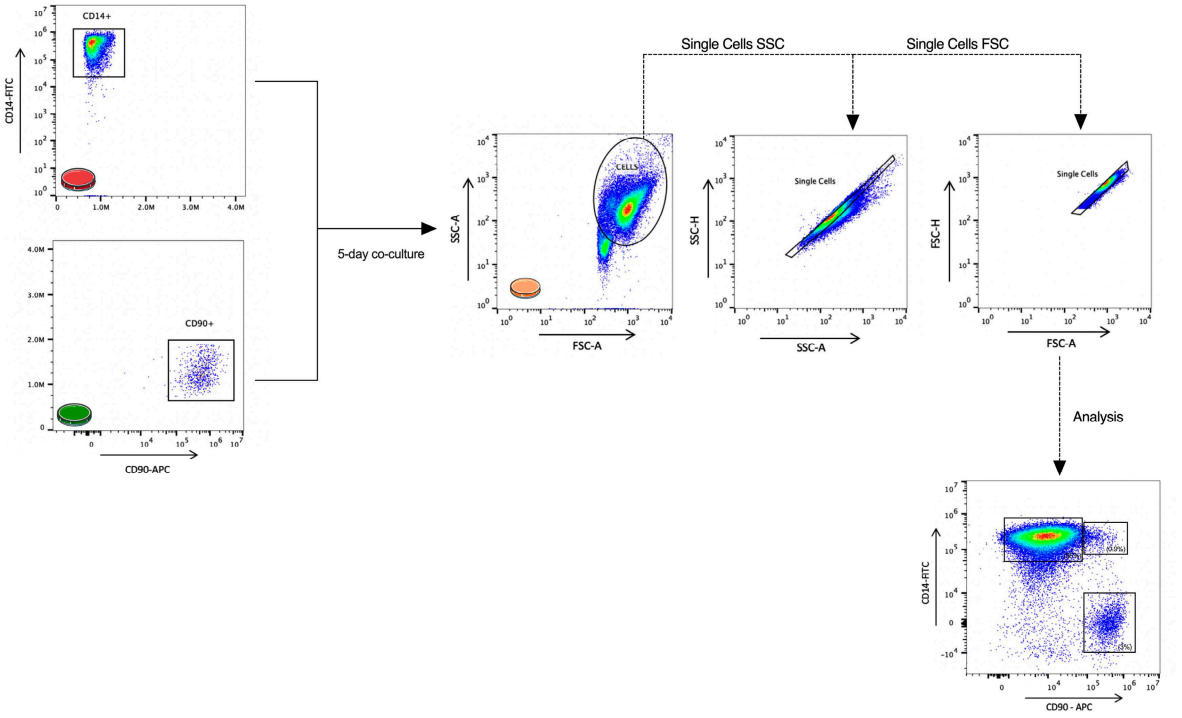

Figure S1: Gating strategy followed for *in vitro* FHC identification (CD14$^+$ monocytes, red; CD90$^+$ hADMSCs, green and CD90$^+$CD14$^+$ FHCs, tangerine).

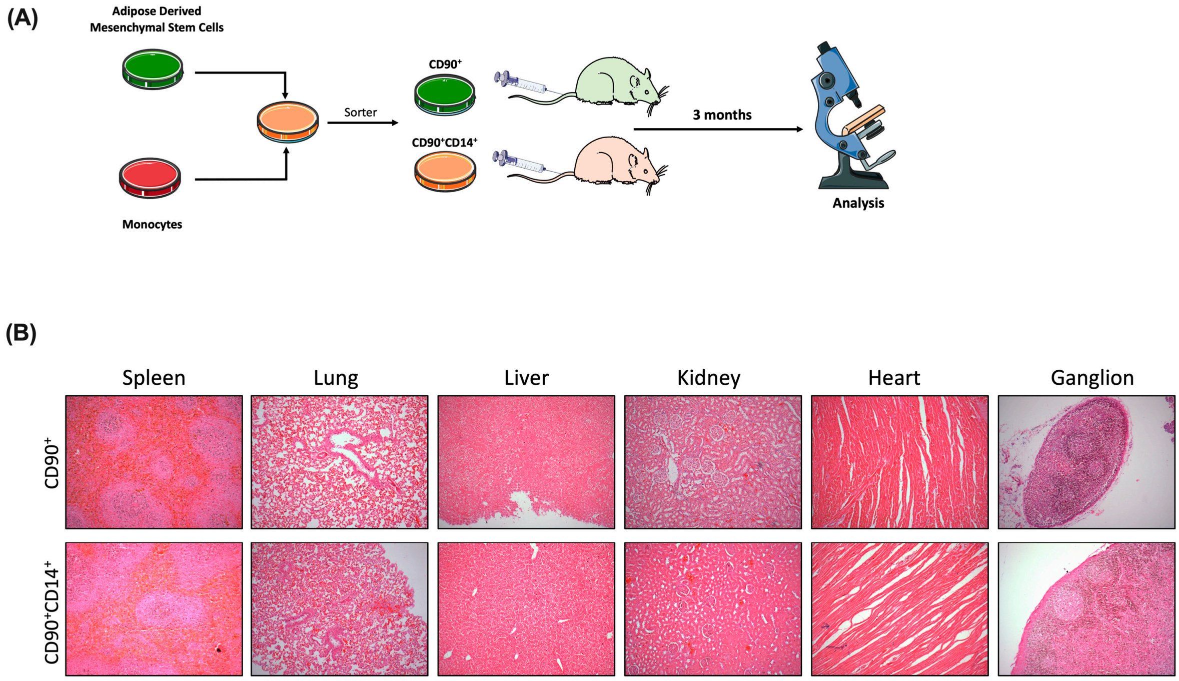

Figure S2: FHCs do not generate tumours *in vivo*. **A**, Schematic representation of experimental conditions for *in vivo* administration of CD90$^+$ hADMSCs (green) and CD90$^+$CD14$^+$ FHCs (tangerine). **B**, Representative Haematoxylin & Eosin staining of different organ tissue sections from sorted CD90$^+$ (upper row) and CD90$^+$CD14$^+$ (lower row) infused rats at 10x magnification.

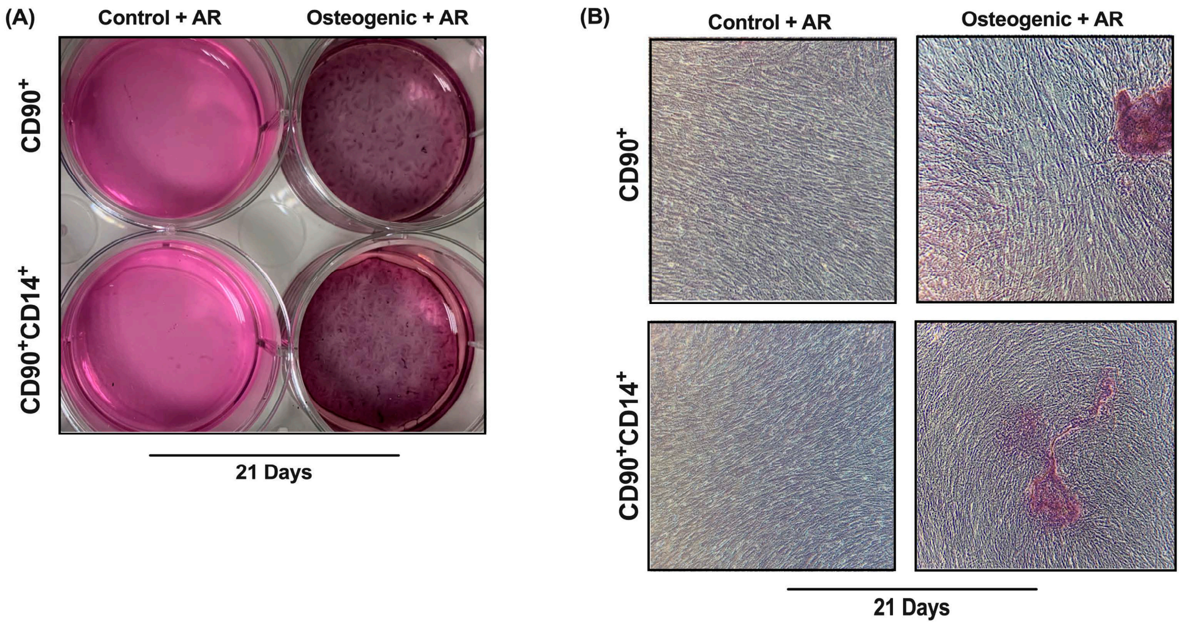

Figure S3: Osteogenic differentiation abilities of sorted hADMSCs and FHCs. **A**, Macroscopic images from sorted CD90+ (upper row) and CD90+CD14+ (lower row) with alizarin red staining to determine osteogenic differentiation. **B**, Representative brightfield microscopy images of cellular differentiation of sorted CD90+ hADMSCs (upper row) and CD90+CD14+ cells (FHCs, lower row) images at 20x magnification.

Table S1: Sequence of primers used for RTqPCR analyses.

Gene	Primer	Sequence (5'-3')
12S	Forward	CCA CGG GAA ACA GCA GTG AT
	Reverse	CTA TTG ACT TGG GTT AAT CGT GTG
cMyc	Forward	GCC AAG CTC GTC TCA GAG AAG
	Reverse	CAG AAG GTG ATC CAG ACT CTG
KLF4	Forward	ACC CAC ACA GGT GAG AAA CC
	Reverse	ATG TGT AAG GCG AGG TGG TC
NANOG	Forward	TGA ACC TCA GCT ACA AAC AGG TG
	Reverse	AAC TGC ATG CAG GAC TGC AGA G
MMP2	Forward	GAT ACC CCT TTG ACG GTA AGG A
	Reverse	CCT TCT CCC AAG GTC CAT AGC
MMP9	Forward	TTC CAA ACC TTT GAG GGC GA
	Reverse	CAA AGG CGT CGT CAA TCA CC
